# Cerebrovascular disease: how serum phosphorus, vitamin D, and uric acid levels contribute to the ischemic stroke

**DOI:** 10.1186/s12883-020-01686-4

**Published:** 2020-03-31

**Authors:** Abolfazl Talebi, Alireza Amirabadizadeh, Samaneh Nakhaee, Zahra Ahmadi, Seyed Mohammad Mousavi-Mirzaei

**Affiliations:** 1 Student Research Committee, Birjand University of Medical Sciences, Birjand, Iran; 2 Cardiovascular Diseases Research Center, Birjand University of Medical Sciences, Birjand, Iran; 3 Medical Toxicology and Drug Abuse Research Center (MTDRC), Birjand University of Medical Sciences, Birjand, Iran

**Keywords:** Stroke, Ischemic, CVA, Phosphorus, Uric acid, Vitamin D, Iran

## Abstract

**Background:**

Associations between serum phosphorus level and the incidence of ischemic stroke are not clear. This study aimed to measure serum phosphorus, vitamin D3, and uric acid levels in ischemic stroke patients compared to a population without ischemic stroke.

**Methods:**

In this cross-sectional study, 133 patients admitted to a neurology ward with the diagnosis of ischemic stroke were compared with a control group comprising 133 age- and gender-matching individuals. The presence of ischemic stroke was confirmed by a neurologist based on clinical signs, symptoms, brain CT scan, and MRI. Blood samples were taken from all patients in the first 24 h of admission to measure serum phosphorus, vitamin D3, calcium, and uric acid levels.

**Results:**

According to the results of this study, uric acid medians in patients with stroke and controls were 4.9 [3.8–6.4] and 3.9 [3.5–4.9] mg/dL, respectively (*p* < 0.001). Median phosphorus and vitamin D levels were significantly lower in stroke patients than the controls (3.6 [3.02–4.21] vs. 4.2 [3.8–4.6]) and (15.1 [8.2–27.9] vs. 22.7 [10.4–39.2]), respectively. Multiple logistic regression analysis showed that the ischemic stroke was positively associated with the vitamin D level and negatively correlated with the uric acid level. The phosphorus level was not significantly predictive of ischemic stroke.

**Conclusion:**

Lower serum levels of vitamin D3 and higher levels of uric acid were associated with ischemic stroke. There are still unknowns about the role of these indicators on ischemic stroke and it requires further studies.

## Introduction

Cerebrovascular accident (CVA) is noted as the second cause of mortality, especially in the elderly population [1]. The main risk factors for CVA are pre-existing heart diseases such as coronary artery disease and atrial fibrillation, hypertension, dyslipidemia, diabetes mellitus, smoking, and 65 years of age or older [2]. Some biochemical factors and circulating molecules have been introduced as risk factors for vascular diseases. For example, a recent experimental study reported higher serum phosphorus levels as a risk factor in the pathogenesis of the vascular disease [3]. AlsoSome other investigations propose the possibility that serum phosphorus concentrations within the reference limit may affect the risk of coronary vascular disease in the general population [4, 5]. In contrast, some other studies report the association between serum phosphorus levels and heart stroke with controversial results [4, 6–9]. However, these studies had no data on the levels of possible mediating factors such as vitamin D levels. Higher serum phosphate has been correlated with increased fibroblast growth factor-23 (FGF-23) activity [10]. Moreover, FGF-23 suppresses serum levels of 1, 25-vitamin D3 by inhibiting the enzyme CYP27B1 (which converts 25-hydroxyvitamin D to 1, 25-dihydroxyvitamin D) [11]. On the other hand, vitamin D insufficiency may activate parathyroid for releasing of parathyroid hormone [12] which was reported to elevate serum uric acid levels [13, 14], although the exact mechanism is not clearly understood.

While the role of vitamin D deficiency and calcium-phosphate abnormalities in the incidence of CAD and CVA has been investigated in some studies, separatly [15–19], to our knowledge, the relation between the serum phosphorous level and other vascular diseases such as ischemic stroke is not well recognized. Accordingly, we investigated serum levels of phosphorus and some possible mediating factors such as calcium vitamin D3, and uric acid all together in patients with ischemic stroke compared to a population without stroke population.

## Materials and methods

The protocol of this case-control study was approved by the Human Research Committee affiliated with Birjand University of Medical Sciences (identifier: ir.bums. REC.1395.117). The participants were recruited from the patients admitted to a referral hospital in eastern Iran (Vali-Asr hospital, Birjand city, South Khorasan province, Iran) between June 2016 to April 2017. A total of 133 patients with ischemic stroke were enrolled as the case group, who were compared with 133 age- and gender-matching subjects without ischemic stroke admitted for cataract surgery as the controls. Participants in the control group had no history of atherosclerotic, thrombotic, or rheumatic vascular diseases. To exclude the embolic causes of stroke, all the patients underwent transthoracic echocardiography and electrocardiogram. All the participants signed an informed consent form. A neurologist confirmed ischemic stroke using radiological modalities, including brain CT scan or brain MRI. All ischemic stroke patients underwent a carotid Doppler ultrasonography using the L5–13 transducer (Medison Accuvix V10 ultrasound system). Blood samples were taken from all patients in the first 24 h of admission to measure serum phosphorus, calcium, uric acid and vitamin D3(OH) levels, with the latter being the best indicator of circulating vitamin D. The severity of stroke on admission quantified by the National Institutes of Health Stroke Scale (NIHSS) to determine the association of different variables with stroke severity.

Serum vitamin D3 was measured by Elisa Reader (ERBA company, Germany) and serum phosphorus, calcium, and uric acid were measured by Auto-analyzer (ERBA company, Germany).

Data analysis was performed using IBM SPSS Statistics software, Version 19 (IBM Corporation, USA). Data were reported as mean ± SD. The Kolmogorov-Simonov test was used to test the normal distribution of numerical variables. The Student T-test or Mann-Whitney test compared the two groups in terms of continuous variables. Spearman correlation coefficient was used to investigate the relationship between quantitave variables. Significant variables (*p*-value < 0.1 in the univariate analysis, T-test, or Mann-Whitney test) were included in the multiple logistic regression model.

## Results

Of the total 266 participants, 51.1% were female, and their mean age was 66.15 ± 11.43 years (range: 40–95 years). Of them, 133 had ischemic stroke and 133 were without ischemic stroke. The mean age of the participants was not statistically different between the two groups (67.52 ± 12.21 vs. 65.78 ± 7.40, *p* = 0.08). In stroke patients, 70 (52.6%) participants were male, and 63 (47.4%) were female; in the control group, 60 (45.1%) were male and 73 (54.9%) were female, with no statistically significant difference between the groups (χ2 = 1.51, *p* = 0.22). The mean NIHSS in stroke patients was 11.47 ± 7.08 with median [inter quartile range] 9.00[5.75–18.00].

Patients in the stroke group showed lower phosphorus and vitamin D levels than the control group (3.6 [3.02–4.21] vs. 4.2 [3.8–4.6], *p* < 0.001) and (15.1 [8.2–27.9] vs. 22.7 [10.4–39.2], *p* = 0.004), respectively. The median [IQR] of uric acid in the case and control groups were 4.9 [3.8–6.4] and 3.9 [3.5–4.9], respectively, with a statistically significant difference between the groups (*p* < 0.001). Calcium in the stroke group was lower than in the control group (9.27 [8.6–9.9] vs. 9.5 [9.1–9.8]); however, the difference was not statistically significant (Table 1).

**Table 1:** Comparison of serum-derived variables in ischemic stroke and control groups

Variables	Total	Ischemic Stroke group	control group	test Result
**Age (years)**	66.65 ± 9.80	67.52 ± 12.21	65.78 ± 7.40	T = 1.26, *p* = 0.08
**Gender**				
**Male**	130(48.8%)	70(52.6%)	60(45.1%)	χ2 = 1.51, *p* = 0.22
**Female**	136(51.2%)	63(47.4%)	73(54.9%)	
**Calcium (mg/dl)**	9.4 [8.9–9.8]	9.27 [8.6–9.9]	9.5 [9.1–9.8]	Z = 1.48, *p* = 0.14
**Phosphorus (mg/dl)**	4.0 [3.4–4.5]	3.6 [3.02–4.21]	4.2 [3.8–4.6]	Z = 5.48, *p* < 0.001
**Uric acid (mg/dl)**	4.4 [3.5–5.6]	4.9 [3.8–6.4]	3.9 [3.5–4.9]	Z = 4.84, *p* < 0.001
**Vitamin D (ng/ml)**	18.3 [9.2–33.5]	15.1 [8.2–27.9]	22.7 [10.4–39.2]	Z = 2.85, *p* = 0.004
**TG (mg/dl)**	114.5[75.7–166.0]	110.5[64.7–163.0]	122.5[84.5–177.5]	Z = 2.23, *p* = 0.03
**FBS (mg/dl)**	89.5[79.7–100.0]	97.0[81.7–125.2]	85.5[76.0–91.7]	Z = 3.84, *p* < 0.001
**LDL (mg/dl)**	111.0[91.0–136.0]	97.0[81.5–119.2]	124.5[103.5–150.2]	Z = 4.59, *p* < 0.001
**HDL (mg/dl)**	38.0[33.0–45.0]	36.5[30.7–42.2]	42.0[34.2–49.7]	Z = 3.40, *p* < 0.001

Values represent medians [interquartile range]

In the case group, there was a positive correlation between vitamin D and phosphorus levels (rho = 0.31, *p* < 0.001). In addition, the phosphorus level had a non-significant correlation with uric acid (rho = 0.06, *p* = 0.64) and calcium levels (rho = 0.03, *p* = 0.78). In the patients without ischemic stroke, there was a negative correlation between calcium and phosphorus levels (rho = − 0.19, *p* = 0.03), phosphorus and uric acid levels (rho = 0.33, *p* = 0.004), and phosphorus and calcium levels (rho = 0.26, p = 0.03).

In the stroke patient group, there was a non-significant negative spearman correlation between phosphorus and right- and left-side carotid intima-media thickness ((rho = − 0.18, *p* = 0.14) and (rho = − 0.05, *p* = 0.67)).

In diabetic patients with stroke, the mean level of phosphorus was significantly lower than in diabetic control group (4.01 ± 0.89 vs. 4.40 ± 0.35, *p* = 0.039).

The relationship of uric acid with TG, LDL, HDL and FBS were not statistically significant in two studied groups (Table 2). In stroke patient, the results spearman correlation showed that there is a significant positive relationship between blood uric acid levels and NIHSS (rho = 0.52, *p* = 0.01). Also, there was a significant negative relationship between phosphorus and NIHSS (rho = − 0.48, *p* = 0.02). But, there was not a significant relationship between VitD levels and NIHSS (rho = 0.19, *p* = 0.41).

**Table 2:** The relationship between uric acid and TG,LDL,HDL,TC and FBS in two groups study

Group	Variable	TG	LDL	HDL	FBS
**Ischemic Stroke**	**Uric acid**	rho = − 0.01, *p* = 0.9	rho = − 0.007, *p* = 0.95	rho = − 0.03, *p* = 0.79	rho =0.13,*p* = 0.27
**Control**	**Uric acid**	rho = − 0.02,*p* = 0.87	rho =0.05,*p* = 0.65	rho =0.06,*p* = 0.63	rho = −0.09,*p* = 0.43

Multiple logistic regression analysis model proposed two factors as significant predictors in stroke patients including vitamin D [OR (95% CI):0.98(0.96–0.99), *p* = 0.007] and uric acid [OR (95% CI):1.48(1.25–1.76), *p* < 0.001] after adjusting for hyperlipidemia, diabetes, hypertension, cigarette smoking, and addiction (Table 3). Based on the results of this study, sensitivity, specificity, and area under the curve for multiple logistic regression model were 60.9, 75.9, and 70.3%, respectively. The Hosmer-Lemeshow goodness of fit test confirmed the model (χ2 = 15.35, *p* = 0.06).

**Table 3:** Multiple logistic regression analysis to predict ischemic stroke as per the studied variables

Variable	B (SE)	OR (95% CI)	*p*-value
**Phosphorus (mg/dl)**	− 0.02 (0.07)	0.97 (0.84–1.13)	0.77
**Uric acid (mg/dl)**	0.39 (0.08)	1.48 (1.25–1.76)	< 0.001
**Vitamin D (ng/ml)**	−0.02 (0.007)	0.98 (0.96–0.99)	0.007

## Discussion

Results of the current study showed that serum phosphorus levels were not significantly predictive of ischemic stroke. Consistent with our results, one meta-analysis covering 32,608 patients from five studies showed that there was no relationship between serum phosphorus level and vascular diseases [20]. Another study with a 3.9-year follow-up of 3437 patients undergoing hemodialysis reported that a lower serum phosphorus level can cause ischemic stroke and that a higher phosphorus level can cause hemorrhagic stroke. The mechanism is that a higher level of phosphorus triggers inflammatory cytokines and ruptures atherosclerotic lesions, similar to mechanisms that cause coronary artery disease (CAD) [19]. Conditions such as end-stage renal disease put patients in higher serum phosphorus level in the long term. An elevated level of serum phosphorus increases inflammatory cytokines [21], while a restricted phosphorus intake can decrease inflammatory cytokines. A follow-up study showed that higher serum phosphorus levels could increase the risk of CAD-associated mortality in patients with chronic kidney disease in CKD- and CAD-free healthy population [22].

Some studies have considered a role for higher serum phosphorus levels in the pathogenesis of calcified coronary atherosclerotic plaques in young adults and the elderly population [23, 24]. Two studies showed that higher serum phosphorus could increase carotid intima-media thickness [25, 26]. However, our study showed that there is a non-significant negative relation between serum phosphorus level and carotid intima-media thickness on both sides of the neck. Overall, these contradictory results propose that the role of serum phosphorus level in the incidence of vascular diseases, especially ischemic stroke, is unclear and warrant further studies.

A major source of serum phosphorus is diet. The kidney is the primary site of serum phosphorus regulation. Traditionally, the parathyroid hormone is assumed to regulate serum phosphorus, calcium, and vitamin D levels; however, recent studies propose a novel system that tightly controls the serum levels of phosphorus, calcium, and vitamin D3 production, namely, FGF-23. Increased serum phosphorus, vitamin D3, or calcium can up-regulate FGF-23 in osteocytes. Finally, it binds to the Klotho/FGFR1c receptor complex in tubular epithelial cells in the kidneys and, in turn, induces the excretion of phosphorus from the body [27, 28]. Moreover, by stimulating 24-hydroxylase, it can decrease vitamin D3 level [27]. As an explanation for lower levels of phosphorus in ischemic stroke patients compared to subjects without ischemic stroke, it should be noted that the emergence of atherosclerotic lesions begins from childhood and that these atherosclerotic lesions develop into calcified lesions with aging [29]. It is probable that the serum phosphorus level increases in the middle age.

On the contrary, counter-regulatory mechanisms such as decreased FGF-23 can reduce serum phosphorus, vitamin D3, and calcium levels [30]. In addition, a lower level of serum phosphorus has been reported in patients with risk factors of stroke, such as hypertension and diabetes. Hypophosphatemia can cause glucose intolerance and tissue insensitivity to insulin [31]. One study revealed that lower serum phosphorus is accompanied by an increased percentage of diabetes mellitus [19]. Our study showed that diabetic patients with stroke had a lower serum level of phosphorus than diabetic patients of the control group.

Based on the results of this study, vitamin D deficiency was associated with ischemic stroke. Our study also proved the results of recent studies regarding the role of vitamin D3 and serum uric acid level in the incidence of ischemic stroke. A large-scale original study on 10,170 healthy individuals with 21 years of follow-up showed that individuals with a lower level of vitamin D3 are at a higher risk of developing ischemic stroke with a hazard ratio of 1.36 [16]. Our study revealed that a lower serum level of vitamin D3 was associated with ischemic stroke at 1.02 odds ratio. The potential mechanism for the association between vitamin D levels and ischemic stroke is likely to be linked with the association between low levels of 25-OH-vitD and traditional risk factors of ischemic stroke such as hypertension, thrombosis, and atherosclerosis [16, 32–34]. Vitamin D3 has an important neuroprotective role through several mechanisms such as induction of the endogen pathway of antioxidant system and suppression of inflammatory cytokines [15]. Also some studies has been reported that the lower levels of 25(OH) D was associated with an increased risk of having elevated serum uric acid which can act as an indirect pathway [28].

Our study showed that patients with a higher serum level of uric acid are at an increased risk of ischemic stroke with 1.48 odds ratio. Uric acid is the result of purine nucleotide metabolism. Recent studies have revealed that uric acid can increase inflammatory cytokines such as C-reactive protein, IL-6, and TNF-α. Therefore, it could have some associations with vascular diseases such as CAD, ischemic stroke, and hypertension [35].

### Study limitations

The associations presented in this study might be confounded by unknown factors. The results from this study are limited by the small sample size, the lack of a cohort to control our findings, lack of vascular risk factor history, lack of data on baseline neuroimaging.

## Conclusion

This study showed that serum phosphorus levels were not significantly predictive of ischemic stroke; however, lower serum levels of vitamin D3 and higher levels of uric acid were associated with ischemic stroke. There are still unknowns about the role of these indicators on ischemic stroke. Based on the study design of our study, we cannot explain definitely that the elevated levels are the risk of ischemic stroke and it requires further studies.

## Data Availability

The datasets used and/or analysed during the current study available from the corresponding author on reasonable request.

## References

[1] Feigin VL, Krishnamurthi RV, Parmar P, Norrving B, Mensah GA, Bennett DA (2015). Update on the global burden of ischemic and hemorrhagic stroke in 1990-2013: the GBD 2013 study. Neuroepidemiology..

[2] Leira Y, Seoane J, Blanco M, Rodriguez-Yanez M, Takkouche B, Blanco J, et al. Association between periodontitis and ischemic stroke: a systematic review and meta-analysis. Eur J Epidemiol. 2017;32(1):43–53. 10.1007/s10654-016-0170-6 27300352

[3] Sitara D, Razzaque MS, Hesse M, Yoganathan S, Taguchi T, Erben RG (2004). Homozygous ablation of fibroblast growth factor-23 results in hyperphosphatemia and impaired skeletogenesis, and reverses hypophosphatemia in Phex-deficient mice. Matrix Biol.

[4] Tonelli M, Sacks F, Pfeffer M, Gao Z, Curhan G (2005). Relation between serum phosphate level and cardiovascular event rate in people with coronary disease. Circulation..

[5] Arking DE, Becker DM, Yanek LR, Fallin D, Judge DP, Moy TF (2003). KLOTHO allele status and the risk of early-onset occult coronary artery disease. Am J Hum Genet.

[6] Aronson D, Kapeliovich M, Hammerman H, Dragu R (2013). The relation between serum phosphorus levels and clinical outcomes after acute myocardial infarction. PLoS One.

[7] Foley RN, Collins AJ, Ishani A, Kalra PA (2008). Calcium-phosphate levels and cardiovascular disease in community-dwelling adults: the atherosclerosis risk in communities (ARIC) study. Am Heart J.

[8] Slinin Y, Blackwell T, Ishani A, Cummings SR, Ensrud KE, Investigators M (2011). Serum calcium, phosphorus and cardiovascular events in post-menopausal women. Int J Cardiol.

[9] Wannamethee SG, Sattar N, Papcosta O, Lennon L, Whincup PH (2013). Alkaline phosphatase, serum phosphate, and incident cardiovascular disease and total mortality in older men. Arterioscler Thromb Vasc Biol.

[10] Antoniucci DM, Yamashita T, Portale AA (2006). Dietary phosphorus regulates serum fibroblast growth factor-23 concentrations in healthy men. J Clin Endocrinol Metabol.

[11] Quarles LD (2012). keletal secretion of FGF-23 regulates phosphate and vitamin D metabolism. Nat Rev Endocrinol.

[12] Emilion E, Emilion R (2011). Estimation of the 25 (OH) vitamin D threshold below which secondary hyperparathyroidism may occur among African migrant women in Paris. Int J Vitam Nutr Res.

[13] Hui JY, Choi JWJ, Mount DB, Zhu Y, Zhang Y, Choi HK (2012). The independent association between parathyroid hormone levels and hyperuricemia: a national population study. Arthritis Res Ther.

[14] Miller P, Schwartz E, Chen P, Misurski D, Krege J (2007). Teriparatide in postmenopausal women with osteoporosis and mild or moderate renal impairment. Osteoporos Int.

[15] Bolland MJ, Bacon CJ, Horne AM, Mason BH, Ames RW, Wang TK (2009). Vitamin D insufficiency and health outcomes over 5 y in older women. Am J Clin Nutr.

[16] Brøndum-Jacobsen P, Nordestgaard BG, Schnohr P, Benn M (2013). 25-Hydroxyvitamin D and symptomatic ischemic stroke: an original study and meta-analysis. Ann Neurol.

[17] Buell J, Dawson-Hughes B, Scott T, Weiner D, Dallal G, Qui W (2010). 25-Hydroxyvitamin D, dementia, and cerebrovascular pathology in elders receiving home services. Neurology..

[18] Pilz S, Dobnig H, Fischer JE, Wellnitz B, Seelhorst U, Boehm BO (2008). Low vitamin D levels predict stroke in patients referred to coronary angiography. Stroke..

[19] Yamada S, Tsuruya K, Taniguchi M, Tokumoto M, Fujisaki K, Hirakata H (2016). Association between serum phosphate levels and stroke risk in patients undergoing hemodialysis: the Q-cohort study. Stroke..

[20] Kazerani H, Rai A, Heidary-Moghadam R, Asgari N, Nowroozi M, Rasouli MH (2014). Relationship between using raw opium and opioids with coronary artery stenosis based on coronary an-giography findings. J Biol Today’s World.

[21] Gutiérrez OM (2013). The connection between dietary phosphorus, cardiovascular disease, and mortality: where we stand and what we need to know. Adv Nutr.

[22] Dhingra R, Sullivan LM, Fox CS, Wang TJ, D’Agostino RB, Gaziano JM (2007). Relations of serum phosphorus and calcium levels to the incidence of cardiovascular disease in the community. Arch Intern Med.

[23] Foley RN, Collins AJ, Herzog CA, Ishani A, Kalra PA (2009). Serum phosphorus levels associate with coronary atherosclerosis in young adults. J Am Soc Nephrol.

[24] Lutsey PL, Alonso A, Michos ED, Loehr LR, Astor BC, Coresh J (2014). Serum magnesium, phosphorus, and calcium are associated with risk of incident heart failure: the atherosclerosis risk in communities (ARIC) study. Am J Clin Nutr.

[25] Ix JH, De Boer IH, Peralta CA, Adeney KL, Duprez DA, Jenny NS (2009). Serum phosphorus concentrations and arterial stiffness among individuals with normal kidney function to moderate kidney disease in MESA. Clin J Am Soc Nephrol.

[26] Ruan L, Chen W, Srinivasan SR, Xu J, Toprak A, Berenson GS (2010). Relation of serum phosphorus levels to carotid intima–media thickness in asymptomatic young adults (from the Bogalusa heart study). Am J Cardiol.

[27] Hardcastle M, Dittmer K (2015). Fibroblast growth factor 23: a new dimension to diseases of calcium-phosphorus metabolism. Vet Pathol.

[28] Bergwitz C, Jüppner H (2010). Regulation of phosphate homeostasis by PTH, vitamin D, and FGF23. Annu Rev Med.

[29] McGill HC Jr., McMahan CA, Herderick EE, Malcom GT, Tracy RE, Strong JP. Origin of atherosclerosis in childhood and adolescence. Am J Clin Nutri. 2000;72(5 Suppl):1307s-15s. 10.1093/ajcn/72.5.1307s 11063473

[30] El-Abbadi MM, Pai AS, Leaf EM, Yang H-Y, Bartley BA, Quan KK (2009). Phosphate feeding induces arterial medial calcification in uremic mice: role of serum phosphorus, fibroblast growth factor-23, and osteopontin. Kidney Int.

[31] DeFronzo RA, Lang R (1980). Hypophosphatemia and glucose intolerance: evidence for tissue insensitivity to insulin. N Engl J Med.

[32] Li YC, Qiao G, Uskokovic M, Xiang W, Zheng W, Kong J (2004). Vitamin D: a negative endocrine regulator of the renin–angiotensin system and blood pressure. J Steroid Biochem Mol Biol.

[33] Mehta V, Agarwal S (2017). Does vitamin D deficiency lead to hypertension?. Cureus..

[34] Ohsawa M, Koyama T, Yamamoto K, Hirosawa S, Kamei S, Kamiyama R (2000). 1α, 25-dihydroxyvitamin D3 and its potent synthetic analogs downregulate tissue factor and upregulate thrombomodulin expression in monocytic cells, counteracting the effects of tumor necrosis factor and oxidized LDL. Circulation..

[35] Kanellis J, Kang D-H (2005). Uric acid as a mediator of endothelial dysfunction, inflammation, and vascular disease. Semin Nephrol.

